# Exploring Task-Specific Independent Standing in 3- to 5-Month-Old Infants

**DOI:** 10.3389/fpsyg.2017.00657

**Published:** 2017-04-28

**Authors:** Hermundur Sigmundsson, Håvard W Lorås, Monika Haga

**Affiliations:** ^1^Department of Psychology, Norwegian University of Science and TechnologyTrondheim, Norway; ^2^Reykjavik UniversityReykjavík, Iceland; ^3^Department of Neuromedicine and Movement Science, Norwegian University of Science and TechnologyTrondheim, Norway

**Keywords:** infants, motor development, task-dependent, independent standing

## Abstract

Perspectives on developmental milestones suggest that an infant’s ability to stand without support occurs at the age of 9–16 months. The two exploratory tasks were part of a baby swimming routine, conducted over a period of 12 weeks (24 sessions), and the aim was to examine whether young infants (mean age 97 days) improved their performance in standing as measured by prolonged time-to-stand. The data suggest that 3- to 5-month-old infants are capable of demonstrating signs of motor learning in task-specific standing. The results appear remarkable when compared to the expected age required for other forms of independent standing. The developmental process of independent standing is discussed in relation to the complex interaction between genetic and environmental factors.

## Introduction

Perspectives on developmental milestones suggest that an infant’s ability to stand without support occurs at the age of 9–16 months (Piper and Darrah, 1994; Claxton et al., 2013). Independent standing is required for vital goal-directed motor behavior like reaching in upright position and walking (Adolph, 2008). However, adopting a bipedal posture is a difficult milestone to reach. Due to their large heads and short feet, infants have a relatively high center of mass (CoM) combined with a relatively small base of support (BoS). In order to achieve this posture and overcome multiple degrees of freedom relative to the force of gravity, direction-specific postural adjustment based on the integration of multisensorial afferent input from somatosensory, visual, and vestibular systems is required (Carpenter et al., 2010).

Motor development is driven by involvement in general physical activity and exercise (Zelazo et al., 1972; Hopkins and Westra, 1988). Similar, experience affects the development of postural adjustment (Hadders-Algra et al., 1996; Sigmundsson and Hopkins, 2009; Claxton et al., 2013). Development of postural adjustment and control is typically characterized by an increase in the infant’s motor repertoire and improved competence in selecting the best strategy to respond to the specifics of the situation, i.e., choosing more advanced anticipatory actions (Claxton et al., 2012; Haddad et al., 2013; Hadders-Algra, 2013). The idea that experience and stimuli serve as a basis for motor development, is mainly derived from perspectives like “neuronal group selection theory” (NGST) (Edelman, 1987, 1992; Thelen and Smith, 1994; Hadders-Algra, 2000; Blauw-Hospers et al., 2011). According to this perspective, the gradual learning of efficient movement to achieve a given task depends on experience and self-produced trials-and-errors (Thelen and Smith, 1994; Hadders-Algra, 2000). Indeed, normally developing infants demonstrate experience-dependent control of standing at a mean age of 11 months (Claxton et al., 2013) albeit a complete description of the developmental process toward this motor milestone has to take into account the dynamical interaction between growth, maturation, learning, and experience (Thelen and Smith, 1994).

**FIGURE 1:**
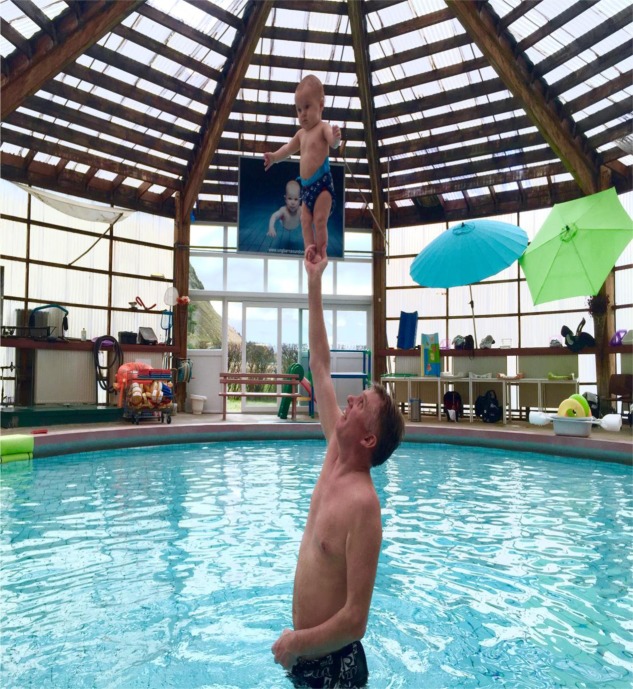
**Example of a 4.6-month-old infant performing the independent standing in the hand of the instructor**.

As stated by Hedberg et al. (2007), there are methodological challenges associated with studying the development of independent standing, as infants under the age of 9 months *per se* are not expected to be able to stand independently. In order to explore independent standing, 3–5 months old infants participating in a baby swimming course were recruited to participate in the study. Choosing this kind of context was mainly based on anecdotal evidence and observations of the instructor that emphasized independent standing as a central task in the water-based program (**Figure 1**) in which infants appeared to demonstrate early signs of motor learning/postural readiness. Also, it was considered as a safe and joyful context for the infant and their parents to study this specific motor behavior. Given the age of the participating infants, indications of motor learning in the standing tasks could imply earlier signs of experience-dependent independent standing than previously reported in the literature (Hadders-Algra, 2005; Claxton et al., 2013). In other words, the study of infants engaging in these tasks enables investigation of task-specific independent standing among putative non-standing infants. Subsequently, the aim of this explorative study on a specific independent standing tasks, conducted as a part of a baby swimming routine over a period of 12 weeks (24 sessions), was to examine whether young infants improved their performance as measured by prolonged time-to-stand.

## Materials and Methods

### Participants

The infants (*N* = 13; 7 girls and 6 boys) and their parents were recruited from the Reykjavik area in Iceland. The parents had signed up for participation in a water-based program. One infant kept falling asleep during the initial sessions, which caused the parents to withdraw from the study. The final sample participating in the study thus consisted of 12 infants (7 girls/5 boys). All were healthy and born at term without complications. The mean (*SD*) age, weight and height upon entering the program was 97 days (13.4), 6.4 (2.6) kg and 62.2 (0.9) cm, respectively. The infant’s score on the Alberta Infant Motor Scale (AIMS) ranged from 9/25th to 15/50th, which indicated a gross motor performance within the normal range. The demographical data of each individual infant can be seen in **Table 1**.

**Table 1:** Demographical data on each individual infant participating in the study.

Subject	Age^1^	AIMS^2^	Gender	Weight (kg)	Height (cm)	PE^3^ (M/F^4^)	Compliance^5^
1	109	13/75th	Female	7.4	64	U/U	15
2	90	12/50th	Male	7.2	65	U/U	19
3	87	13/50th	Female	5.3	60	U/U	18
4	100	10/25th	Female	6.7	63	U/U	21
5	94	13/50th	Male	6.8	64	U/U	19
6	119	15/50th	Male	6.3	65	H/P	17
7	111	14/50th	Male	5.7	62	H/P	11
8	83	9/25th	Female	6.0	61	U/U	21
9	83	13/75th	Female	6.5	60	U/U	18
10	116	13/25th	Male	8.0	65	U/U	16
11	88	12/75th	Female	4.9	57	U/U	18
12	85	10/25th	Female	6.0	60	U/U	22


*^1^Number of days old when entering the study.*

*^2^Alberta Infant motor scale score.*

*^3^Parents education: P: primary school; H: High school; U: University.*

*^4^M/F: mother/father.*

*^5^Number of sessions each individual infant participated in.*

### Procedure

The infants and at least one of their parents participated in the water-based activities for maximum 24 sessions (two times a week over 12 weeks). At the first session, a trained physiotherapist assessed the gross motor development while the parents completed a brief questionnaire. The program were implemented in a facility designed for water-based activities for children, consisting of an indoor circular pool with a radius of 5 m, high temperature (34°C in the water and 26°C in the air), and shallow water (depth ranging from 0.8–1.2 m). During all sessions, the infants and their parents were accompanied by an instructor in the water. The sessions lasted for 1 h. The study was conducted in accordance with the Helsinki Declaration. Ethical approval for this study was granted by the Icelandic Data Protection Authority. All parents (or guardians) received extensive written descriptions of the goals of the study. Written informed consents were obtained before the children attended the study.

### Independent Standing Tasks in the Water-Based Program

**FIGURE 2:**
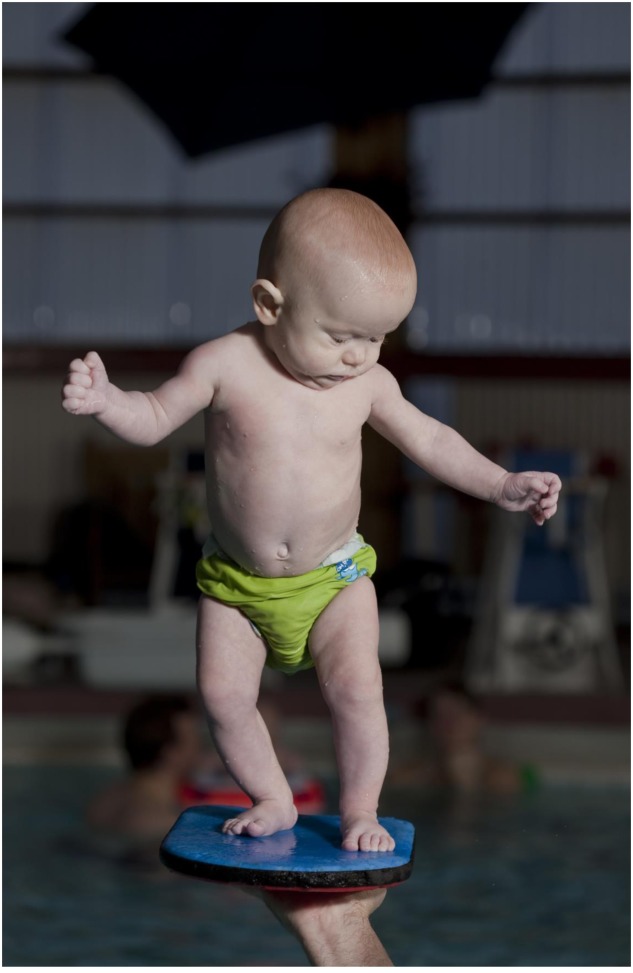
**Example of a 4.8-month-old infant performing independent standing task on a corkboard held by the instructor**.

As this was not a study on water-based activities for infants in general, only a short description of the specifics of an entire baby-swimming session will be provided: Initially, a typical session lasted for 1 hour and consisted of a mixture of gross and fine motor activities. It started with a ‘warm-up’ stage in which the instructor and parents sang a song for the children while moving them slowly through the water. Next, the instructor assisted the infants in performing somersaults on a thin mattress floating on the water and diving under water. They were also encouraged to pick up rings from the water and jump into the water from a supported position along the side of the pool. During these manual exercises, the infants were positioned within reaching distance of objects floating on the water, allowing them to reach and grasp for the objects. In the independent standing tasks examined in this study, the instructor lifted the infants one-by-one to enable them to practice independent standing for a maximum of 15 s. This latter time limit was initiated for practical reasons, i.e., manageable for infants (e.g., attention) and instructor (e.g., fatigue, as it is strenuous for shoulder and arm muscles to hold an infant in this position) and also considered a long enough time period for demonstrating changes in task performance. These latter tasks either consisted of independent standing in the hands of the instructor (**Figure 1**), or standing on a corkboard held up by the instructor (**Figure 2**).

### Tasks and Measures

#### Parental questionnaire

The parental questionnaires included items on the respective infants’ gender, weight, height (at project start), and educational level of the parents.

#### Gross motor development

Alberta Infant motor scale (AIMS) (Piper and Darrah, 1994) was applied to assess the infant’s gross motor function at baseline. AIMS is a norm-referenced measure of gross motor development in infants from birth through independent walking. AIMS has high degrees of test–retest, intra-rater, and inter-rater reliability when administered on normally developing full-term infants (Piper and Darrah, 1994).

#### Video analysis

All sessions (*n* = 24), as the infants and at least one of their parents participated in the pool activities along with an instructor, were recorded by a video camera operating at 60 Hz (Canon Legria HFR506) mounted on a tripod positioned 2 m from the pool. This allowed for a complete view of the behavior of the infants during the standing tasks (in-hand or on a corkboard). The 24 h of video files were examined frame-by-frame by a project assistant blinded of the aim of the study. The variable measured in the two standing tasks was time-to-stand operationalized by establishing the time point in which the instructor removed truncus support with the left hand and the time elapsed when infants demonstrated signs of knee/hip flexion and/or initiated truncus support from the instructor.

#### Statistical analysis

The association between baseline measures and the number of sessions each individual infant participated in before reaching the 15-s mark during the independent standing tasks was examined using Spearman’s rho correlations. The cumulative increase in the number of infants achieving 15 s of independent standing throughout the 12-week study was examined by linear regression. All statistics were analyzed with IBM SPSS Statistics 23.0 with *p* < 0.05 as a statistical significance criterion.

## Results

Results of the correlation analysis between the number of sessions each individual infant participated in before reaching 15 s of independent standing indicated no significant associations against baseline measures: *age* (*ρ* = 0.34 and *p* = 0.30), *AIMS score* (*ρ* = 0.39 and *p* = 0.23), *AIMS percentile* (*ρ* = 0.42 and *p* = 0.20), *weight* (*ρ* = 0.56 and *p* = 0.07), *height* (*ρ* = 0.32 and *p* = 0.34).

**FIGURE 3:**
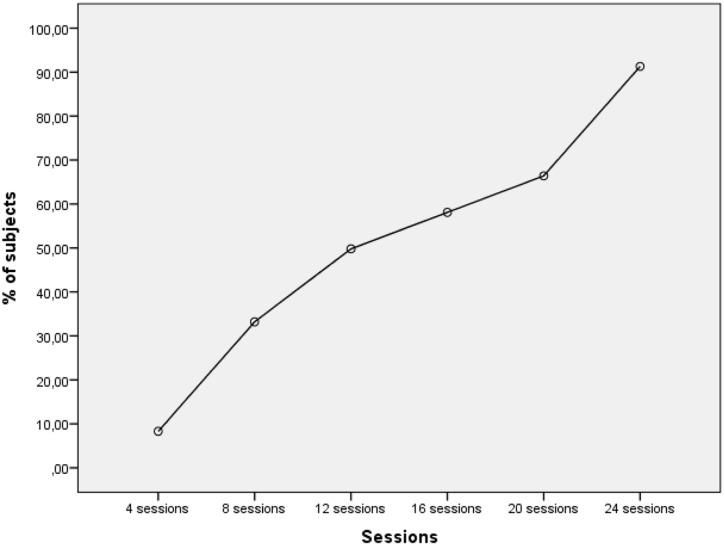
**Proportion of infants able to stand in the hand of the instructor (≥15 s) across the 12-week study period**.

Across the 12-week period of water-based activities for two times a week, there was a significant association between number of sessions conducted and the cumulative increase in infants achieving 15s of independent standing in the instructors hand (*r*^2^ = 0.97 and *p* < 0.001) (**Figure 3** and **Table 2**) and independent standing on the cork board (*r^2^* = 0.92 and *p* < 0.001).

**Table 2:** Individual data of time in standing (in seconds) in the hand of the instructor, showing in which week (session 1 or 2) the child was able to stand for 15 s or more, and the infants age at that point.

		Week (W)											
		
	Subject/Session 1 or 2/Age	W 1	W 2	W 3	W 4	W 5	W 6	W 7	W 8	W 9	W 10	W 11	W 12
1	Session 1	4.9											
	Session 2	15											
	Age^∗^	3.7 m^∗^											

2	Session 1	0	4.52	–	0	0.37	2.83	5.05	3.36	–	0.87	5.08	–
	Session 2	3.87	5.04	0.58	0	0	–	6.20	–	4.23	1.75	8.27	5.87

3	Session 1	0	–	0	0	0	0	6.16	–	–	8.36	–	
	Session 2	0	0	0	0	0.53	2.42	10.45	10.99	–	–	15	
	Age^∗^											5.3 m^∗^	

4	Session 1	–	0	0	0	0	0.50	–	8.82	13.57			
	Session 2		–	0	0	0	0.72	8.61	5.59	22.88			
	Age^∗^									5.3 m^∗^		

5	Session 1	1.0	2.51	22.58									
	Session 2	0	6.92										
	Age^∗^			3.6 m^∗^									

6	Session 1	0	0	0	–	0	0	–	–	1.30	8.22	16.10	
	Session 2	0	0	–	0	0	1.66	4.71	–	5.59	6.02		
	Age^∗^											6.3 m^∗^	

7	Session 1	0.63	2.63	2.58									
	Session 2	1.03	2.87	19.86									
	Age^∗^			4.2 m^∗^									

8	Session 1	0	0	0	0	0	2.22	6.12	–	–	10.29	8.67	2.23
	Session 2	0	0	0	0	5.08	11.62	9.57	11.29	10.59	–	12.59	16.75
	Age^∗^												5.4 m^∗^

9	Session 1	1.03	6.19	–	–	17.19							
	Session 2	0.98	6.12	12.24	–								
	Age^∗^					3.7 m^∗^							

10	Session 1	0	1.13	19.83									
	Session 2	0	–										
	Age^∗^			4.3 m^∗^									

11	Session 1	1.27	2.09	7.63	–	–	13.51						
	Session 2	0.91	9.33	5.73	0	12.24	22.57						
	Age^∗^						4.2 m^∗^						

12	Session 1	0	0	0	0	4.34	–	14.57	19.77				
	Session 2	0	0	0	0	14.07	14.66	12.57					
	Age^∗^								4.4 m^∗^				


*0, not able to stand; –, did not participate; ^∗^, age in months when able to stand 15 s.*

During the second session, in the first week of the program, one infant (see subject 1 in **Table 2**) demonstrated the ability to stand in the hand of the instructor for 15 s whereas 50% of them were able to do so after session 12 (during the sixth week). 11 out of 12 children (92%) were able to stand for more than 15 s in the hand of the instructor during this 12-week period. 1 of the infants (subject 2) stood for 8 s (**Table 2**). Furthermore, all infants were able to stand ≥8 s at a mean age of 4.2 months (range 3.2–6.1). Analysis of the time-to-stand on a corkboard reflected a similar pattern: 10 of the 12 children were able to stand for more than 15 s on a corkboard held by an instructor by the end of the 12-week period. Two of the children were not able to stand for more than 2 and 9 s, respectively. Eleven of the infants were able to stand independently ≥8 s. These results occurred independently of initial age, gender, height, weight and AIMS score, as well as the parents’ educational level.

## Discussion

The data suggest that 3- to 5-month-old infants are capable of demonstrating signs of motor learning in task-specific independent standing. The results appear remarkable when compared to the expected age required for other forms of independent standing, e.g., previous data indicate that the emergence of independent standing occurs between the age of 9 and 16 months, while standing up alone when holding on to furniture takes place at the age of 6–12 months (Haywood and Getchell, 2014).

According to Bernstein (1967) learning could be seen as the process of freezing and releasing the degrees of freedom of the body. For infants it is challenging to control and coordinate the body’s degrees of freedom (ankle, knee, hip, and trunk) while maintaining their CoM within the BoS in standing (Claxton et al., 2012).

As observed, the infants in this sample adopted a stiff posture in the standing position (**Figures 1**, **2**), thereby one could speculate that this indicated decreasing the degrees of freedom. This is a common strategy when learning new skills, and can be seen among both infants and older children (Thelen et al., 1993; Harbourne and Stergiou, 2003). It could be suggested that the infants go from freezing to releasing the degrees of freedom (Bernstein, 1967). In other words, they freeze the degrees of freedom to acquire a skill before further experience and training enable them to release the degrees of freedom. This makes it possible to explore the dynamics of a new motor skill in the environment (Harbourne and Stergiou, 2003). Altogether, this could result in increased movement flexibility and improved postural control, while explaining the longer time required for task-specific standing in our sample throughout the course.

The adaptation to increased movement flexibility and release degrees of freedom lends support to Hadders-Algra (2005, 2013), suggesting that more adaptive postural responses emerge with increased experience and higher age. Accordingly, the findings of increased time-to-stand could be a result of an adaptation to more advanced direction specific adjustment (Hadders-Algra, 2005; Hedberg et al., 2007). This phenomenon has earlier been highlighted in studies focusing on postural control in sitting (Hadders-Algra et al., 1996) and standing with and without support (Hedberg et al., 2007). As Hadders-Algra (2010) highlight: the primary genetic determinations is only the starting point for epigenetic cascades, allowing for abundant interaction with the environment and activity-dependent processes (p. 1825). In this respect, Hedberg et al. (2005) found that infants already at the age of 1 month were able to select a specific set of postural behavior, indicating the innate quality of these strategies. If the basic developmental principles of postural adjustment during standing are similar to those of adjustment during sitting (Hedberg et al., 2005) this could also explain the unexpected finding of standing among putative non-standing infants.

In fact, the findings could reflect the continual complex interaction between genetic information and environmental influences that characterize the developmental process, as underlined in the NGST (Hedberg et al., 2007; Ulrich, 2010; Blauw-Hospers et al., 2011). Due to an innate activation of muscles that takes place before independent standing develops (the phase of primary repertoire) (Hadders-Algra, 2000), infants could display postural responses that enable them to stand in the hands of the instructor. Moreover, during the program the infant explores the dynamics of the skill (Blauw-Hospers et al., 2011; Claxton et al., 2012), and development of standing proceeds with the selection of neural network involved on the basis of afferent information produced by behavioral experience (the phase of secondary repertoire) (Hadders-Algra, 2000).

This study has some limitations. Because of the small sample size the correlations should be interpreted cautiously. Moreover, Independent standing requires at least two capabilities: (i) extend the body in the upright position and resist the pull of gravity and (ii) maintain the CoM within the BoS. Regarding point one the practice effect described in the current study may simply facilitate the extension response. Amiel-Tison et al. (2006) noted that antigravity straightening is present in the newborn as a “tactile reflex”, but disappears completely between 4 and 7 months of age as a bias toward flexor tonus increasingly dominates, only to reappear as a self-initiated pattern when the child is able to stand independently. This fits into the perspectives of NGST as in the early phases of development the role of the genome dominates and in later stages the environment and experience become of high importance (Hadders-Algra, 2010, p. 1825). With regard to the second point, it has to be noted that the instructor can assist the infants in keeping the BoS under the CoM in order to maintain equilibrium. This might explain that an infant is capable of conduction the standing tasks (in the hand of the instructor or on the corkboard). However, it might not provide a complete explanation for the observed progression in the tasks during the course of the study. The instructors adjustments has to interact with the infants movements, even though (as raised in the previous point) at least part of the potential practice effect observed in this study might be due to a facilitated extension response. Thus, there might be aspects of dynamical instructor–infant interactions that improves with practice and contributes to increased time-to-stand in the two tasks. One possibility in this regard is that the infants demonstrate a mixture of voluntary (self-generated) and involuntary (instructor-generated) movements, in which the resultant behavior contains reflexive elements. However, there is no sharp distinction between motor actions that appear voluntary vs. involuntary (Stenner and Haggard, 2017), and further study should thus investigate the relative contribution of reflexive or endogenous components in the infants postural movements.

The presented findings should encourage further in-depth studies into the mechanisms behind development of postural strategies in very young and putative non-standing infants. The results of this study; however, needs to be evaluated against the dynamical sharing of motor adjustments between the instructor and the infants in the standing tasks. This requires methodological rigor with assessment of kinematical properties and muscle activity in order to elucidate the interaction between changes in task performance and postural strategies.

## Author Contributions

HS, HL, and MH involved in planning, data collection, data analysis, and writing of the paper.

## Conflict of Interest Statement

The authors declare that the research was conducted in the absence of any commercial or financial relationships that could be construed as a potential conflict of interest.
